# Heterozygous *De Novo* and Inherited Mutations in the Smooth Muscle Actin (*ACTG2*) Gene Underlie Megacystis-Microcolon-Intestinal Hypoperistalsis Syndrome

**DOI:** 10.1371/journal.pgen.1004258

**Published:** 2014-03-27

**Authors:** Michael F. Wangler, Claudia Gonzaga-Jauregui, Tomasz Gambin, Samantha Penney, Timothy Moss, Atul Chopra, Frank J. Probst, Fan Xia, Yaping Yang, Steven Werlin, Ieva Eglite, Liene Kornejeva, Carlos A. Bacino, Dustin Baldridge, Jeff Neul, Efrat Lev Lehman, Austin Larson, Joke Beuten, Donna M. Muzny, Shalini Jhangiani, Baylor-Hopkins Center for Mendelian Genomics, Richard A. Gibbs, James R. Lupski, Arthur Beaudet

**Affiliations:** 1 Department of Molecular and Human Genetics, Baylor College of Medicine, Houston, Texas, United States of America; 2 Texas Children's Hospital, Houston, Texas, United States of America; 3 Institute of Computer Science, Warsaw University of Technology, Warsaw, Poland; 4 Department of Pediatrics, Medical College of Wisconsin, Milwaukee, Wisconsin, United States of America; 5 Children's Clinical University Hospital, Riga, Latvia; 6 Department of Pediatrics, Baylor College of Medicine, Houston, Texas, United States of America; 7 Department of Genetics, Children's Hospital Colorado, Aurora, Colorado; 8 Human Genome Sequencing Center, Baylor College of Medicine, Houston, Texas, United States of America; Stanford University School of Medicine, United States of America

## Abstract

Megacystis-microcolon-intestinal hypoperistalsis syndrome (MMIHS) is a rare disorder of enteric smooth muscle function affecting the intestine and bladder. Patients with this severe phenotype are dependent on total parenteral nutrition and urinary catheterization. The cause of this syndrome has remained a mystery since Berdon's initial description in 1976. No genes have been clearly linked to MMIHS. We used whole-exome sequencing for gene discovery followed by targeted Sanger sequencing in a cohort of patients with MMIHS and intestinal pseudo-obstruction. We identified heterozygous *ACTG2* missense variants in 15 unrelated subjects, ten being apparent *de novo* mutations. Ten unique variants were detected, of which six affected CpG dinucleotides and resulted in missense mutations at arginine residues, perhaps related to biased usage of CpG containing codons within actin genes. We also found some of the same heterozygous mutations that we observed as apparent *de novo* mutations in MMIHS segregating in families with intestinal pseudo-obstruction, suggesting that *ACTG2* is responsible for a spectrum of smooth muscle disease. *ACTG2* encodes γ2 enteric actin and is the first gene to be clearly associated with MMIHS, suggesting an important role for contractile proteins in enteric smooth muscle disease.

## Author Summary

In 1976, a radiologist, Walter Berdon described a group of patients with a rare intestinal and bladder disorder in which the smooth muscle of those organs failed to contract. These patients are unable to digest food, require multiple abdominal surgeries and are diagnosed with megacystis-microcolon-intestinal hypoperistalsis syndrome (MMIHS). Since the description of MMIHS, the genes that cause it have remained a mystery. We followed and obtained DNA from patients with this disorder over a period of over 14 years and assembled a large group of cases. We used whole-exome sequencing, a powerful tool used to identify disease genes, and found mutations in *ACTG2*, a visceral actin gene. Actins are components of muscle contractile units, and one Finnish family has been previously found with less severe gastrointestinal problems due to mutations in this gene. In our patients, we find *de novo* mutations in the majority of cases of MMIHS. However, we also find families with the disease over several generations due to these same mutations. This work provides the first disease gene for MMIHS, and suggests new treatment options.

## Introduction

Berdon first described patients with a severe phenotype characterized by smooth muscle functional failure in 1976, at a time when total parenteral nutrition (TPN) was becoming common clinical practice [1]. Berdon noted that because the functional intestinal defect could not be corrected, finding the cause of the disorder described as megacystis-microcolon-intestinal hypoperistalsis syndrome (MMIHS, OMIM 249210) would be necessary to avoid keeping patients with the disorder as “prisoners” of TPN without otherwise effective treatments. For thirty years, genetic, pathologic, endocrine, and physiologic studies have sought to determine the cause of MMIHS without success, and the clinical history of patients with these disorders often fulfills Berdon's prediction as patients remain on long-term TPN, and the underlying etiology remains unknown.

Clinically, MMIHS is characterized by prenatal bladder enlargement, neonatal functional gastrointestinal obstruction, and chronic dependence on total parenteral nutrition (TPN) and urinary catheterization [2]–[4]. Patients undergo repeated abdominal surgeries, suffer hepatic complications from TPN, and are susceptible to poor nutrition, as well as infectious complications of ileostomies and intravenous and urinary catheters. The first challenge in understanding the genetics of MMIHS has been in characterizing the clinical phenotype. MMIHS is part of a phenotypic spectrum that includes intestinal pseudo-obstruction [5] (OMIM 155310, 609629), hollow visceral myopathy [6], [7] (OMIM 609629), pseudo-Hirschsprung disease [8], and irritable bowel syndrome [9]. Functional gastrointestinal obstruction is also frequently observed associated with other abnormalities such as prune-belly syndrome (OMIM 100100), external ophthalmoplegia (OMIM 277320), and Barrett esophagus (OMIM 611376). However, there is uncertainty about the extent to which locus heterogeneity and variation in expression underlie this clinical variability [9]. In addition, a number of single case reports have proposed an association of MMIHS with other disorders such as trisomy 18 [10], cardiac rhabdomyomas [11], and deletion of 15q11.2 [12]. However, in these cases it is unclear whether these genetic disorders are related to MMIHS or are coincidental findings. Autosomal recessive inheritance of MMIHS (OMIM 249210) has been suggested in numerous cases based on the presence of two affected siblings [3], [13], [14], consanguinity [15] or both [16]–[19], but no genes have been identified to date, although in retrospect a report of a dominant mutation in the *ACTG2* enteric actin gene in a Finnish family with adult onset visceral myopathy has proved to be relevant [20].

Pathologic studies of intestine in MMIHS have similarly been inconclusive [4]. Some studies demonstrate abnormalities of the circular and longitudinal layers of the *muscularis propria* [21], [22], while others focus on abnormalities of ganglion cells, including reduced [4], increased [4], hypertrophic [23], immature, or dysplastic ganglia [4], [19]. An imbalance between intestinal hormones in cases of MMIHS has also been noted [24]. Finally, the intrinsic pacemaker cells of the gastrointestinal tract, the interstitial cells of Cajal, were noted to display abnormalities in MMIHS [25], [26]. Given this broad range of findings, there has been controversy over which pathological changes in the gastrointestinal tract are primary versus secondary [4].

Additional insight into the genetic basis of MMIHS appeared to come from a mouse model of the disease. Mice lacking expression of the α3 subunit of the neuronal nicotinic acetylcholine receptor encoded by the *Chrna3* gene and mice lacking both the β2 and β4 subunits encoded by the *Chrnb2* and *Chrnb4* genes, respectively, displayed megacystis, failure of bladder strips to contract in response to nicotine, widely dilated ocular pupils, growth failure, and perinatal mortality [27], [28]. These subunits are expressed in various sympathetic and parasympathetic ganglia, and lack of transmission at these ganglia could explain the lack of contraction of involuntary smooth muscle. A role for the α3 subunit was further suggested when reduced mRNA levels were measured by *in situ* hybridization, and reduced immunostaining for protein was possibly found in tissues from MMIHS patients [29]. However, antibodies against the neuronal nicotinic receptor subunits are notoriously unreliable [30] and a specific search for mutations in *CHRNA3* and *CHRNB4* in many of the patients studied herein did not identify any potential disease-causing mutations [31].

## Results

### Whole-Exome Sequencing in an MMIHS Cohort

**Figure 1:**
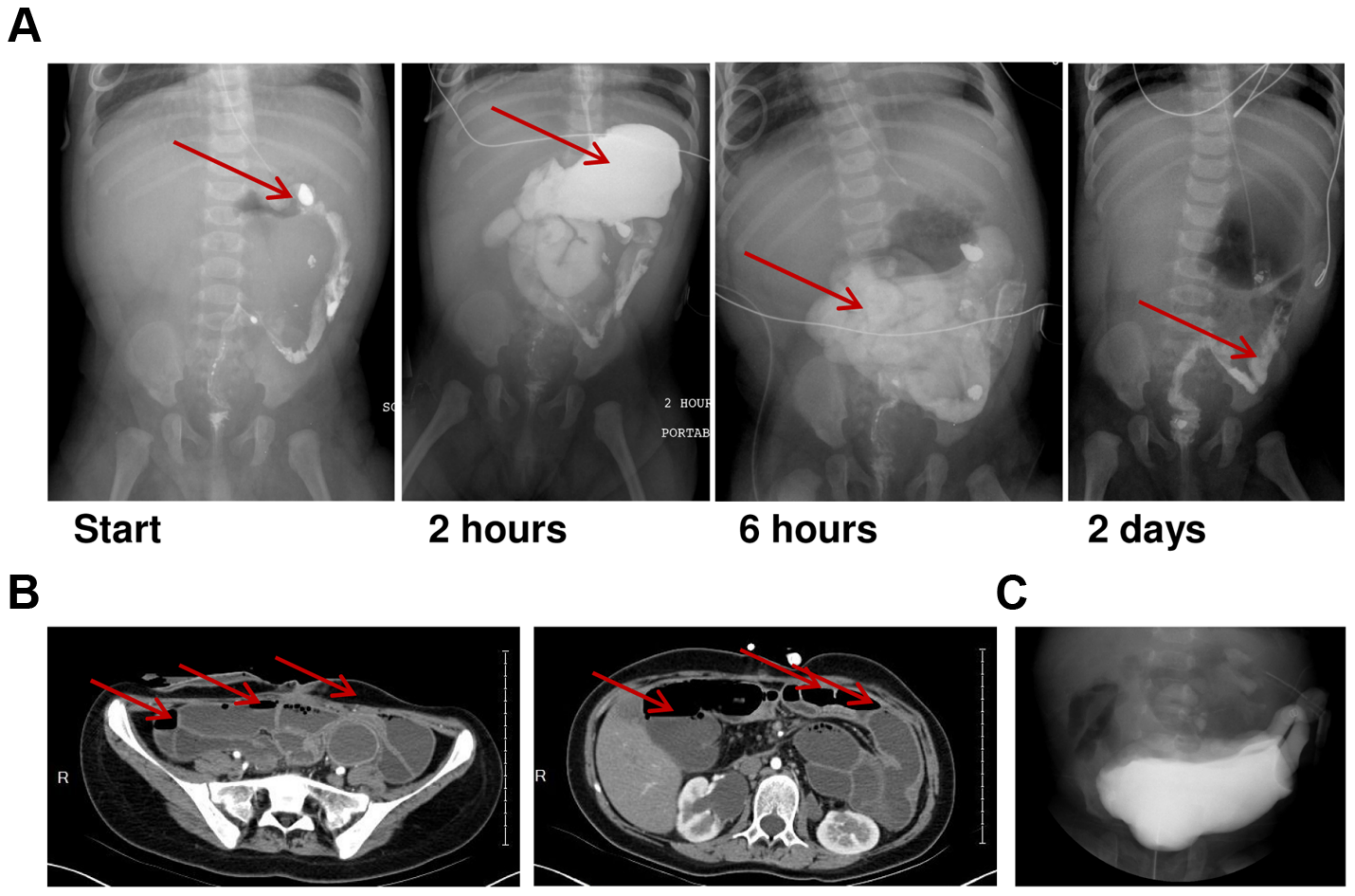
Radiologic features of MMIHS due to *de novo ACTG2* mutations. A.) An infant subject from our cohort (Fam28-1) underwent an upper GI study with small bowel follow-through. Contrast was administered beyond the pylorus (left panel, arrow) in this patient who had undergone a previous Ladd procedure. At two hours, the contrast has mostly passed retrograde back into the stomach (arrow) contrary to expectations. At six hours, the contrast is in the small intestine (arrow) and has not reached the colon. At two days, contrast material is still present in the colon (arrow) suggesting a severely delayed transit of material in the GI tract consistent with hypoperistalsis. No physical obstructive lesions are visualized. B.) An abdominal CT scan on an adolescent patient with MMIHS (Fam26-1). The patient has a diverting ileostomy, but multiple distended bowel loops with air-fluid levels (arrows) are seen at the level of the sacrum (left) and lumbar spine (right). C.) A voiding cystourethrogram on an infant male subject (Fam29-1) showing a grossly distended bladder.

Since the findings in mice harboring mutations in *Chrna3* or in *Chrnb2* and *Chrnb4* combined caused MMIHS-like phenotypes, we have conducted a study of MMIHS aimed at identifying the genetic cause. We collected samples from patients with MMIHS and related phenotypes, some of whom have been previously reported [31]. Our cohort of 34 families to date includes 27 DNA samples from probands including individuals diagnosed with MMIHS (20 probands) as well as intestinal pseudo-obstruction (4 probands), prune belly syndrome (2 probands), and hollow visceral myopathy (1 proband). Examples of radiologic findings are shown in Figure 1. Study recruitment has taken place over a period of 14 years.

We undertook whole-exome sequencing in 11 unrelated probands. The exome sequencing characteristics are summarized in Table 1. Of the 11 probands, eight were diagnosed with MMIHS and three diagnosed with intestinal pseudo-obstruction. We identified heterozygous missense variants in the *ACTG2* gene encoding γ2 enteric actin in six of the 11 individuals. We reasoned that *ACTG2* was an excellent candidate for MMIHS as a thin filament protein in the sarcomere involved in muscular contraction. We therefore undertook Sanger sequencing of all the exons and intron-exon boundaries of *ACTG2* in 16 additional probands in our cohort.

**Table 1:** Exome analysis summary for six probands with MMIHS due to *ACTG2* mutations.

Subject	Total # unfiltered variants	Average Coverage	% at 10× coverage	% at 20× coverage	%at 40× coverage
Fam4-1	129,171	118	95.1	93.3	86.0
Fam19-4	122,121	80	93.1	89.4	75.2
Fam25-1	146,726	112	94.1	91.9	84.1
Fam28-1	131,113	132	92.5	89.5	81.9
Fam29-1	132,740	140	92.9	90.2	83.2
Fam30-1	127,370	135	93.0	90.3	83.1

### *De Novo* and Inherited *ACTG2* Mutations in the MMIHS Cohort

**Figure 2:**
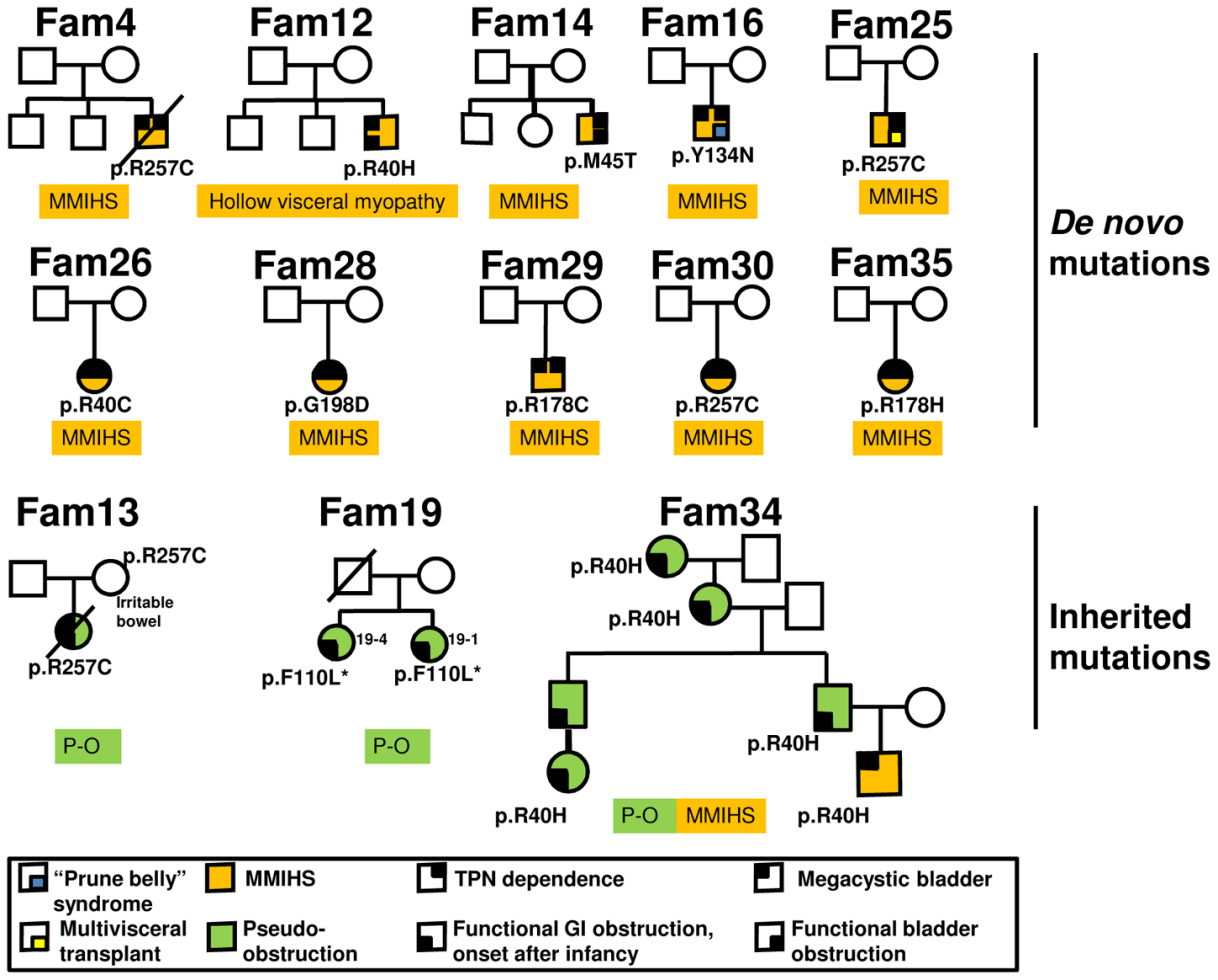
Clinical features and inheritance of *ACTG2* mutations in *de novo* and familial cases. Family pedigrees with clinical features correlated with the severity of smooth muscle dysfunction are shown. The most severe features of TPN dependence and megacystic bladder noted as dark squares within the upper quarters. The diagnosis of MMIHS (orange) was made in all but one subject with *de novo ACTG2* mutations. One subject (Fam 12-1) was diagnosed with gastrointestinal hollow visceral myopathy but had megacystis prenatally. Three families exhibiting dominant inheritance patterns are depicted below. One subject (Fam 13-1) suffered from a megacystic bladder but had later onset functional GI pseudo-obstruction. Another family (Fam19) is shown with two affected siblings with functional GI obstruction. Both carry a nonsynonymous mutation in alternate exon 4 of a predicted short *ACTG2* isoform (Uc010fex.1 indicated by *). Another family exhibited more extensive dominant inheritance (Fam34) consistent with familial visceral myopathy. Multiple paternal relatives suffer from episodes of gastrointestinal obstruction, constipation, gastrointestinal dysmotility, and bladder dysmotility segregating with the same mutation.

The results for all the heterozygous *ACTG2* variants in our cohort are summarized in Table 2. All of these variants were unique to our cohort, as none of the *ACTG2* variants found in our patients, were present neither within the 1000 Genomes project data (http://browser.1000genomes.org/index.html) nor within the NHLBI Exome Sequencing Project (http://evs.gs.washington.edu/EVS/). In addition, within our internal data, excluding the cases presented here, none of these variants were found in approximately 1900 other samples analyzed by the Baylor-Hopkins Center for Mendelian Genomics (http://www.mendelian.org/) nor within 1200 clinical samples analyzed in the BCM clinical laboratory. We did identify a number of other novel heterozygous variants, but these were all distinct from the variants seen in our MMIHS cohort (Table S1). Within our group, 15 probands had mutations in *ACTG2* in comparison to 12 probands in the cohort without mutations in *ACTG2*. Of note, we observed ten apparently *de novo* events (Figure 2). We observed 6 novel C>T transition mutations at CpG dinucleotides affecting arginine amino acid residues including a recurrent mutation (c.769C>T; p.R257C) seen in 3 d*e novo* cases (Fam4-1, Fam30-1, Fam25-1).

**Table 2:** Characteristics of the *ACTG2* mutations in the MMIHS cohort.

Subject	Position (hg19) Chr2	cDNA change^a^	Amino-acid change^c^	CpG	Inheritance	Individuals with variant out of 1900^d^
Fam4-1	74141962	c.769C>T	p.R257C	+	*De novo*	0
Fam11-1	74140693	c.533G>A	p.R178H	+	Maternal	0
Fam12-1	74128450	c.119G>A	p.R40H	+	*De novo*	0
Fam13-1	74141962	c.769C>T	p.R257C	+	Maternal	0
Fam14-1	74129494	c.134T>C	p.M45T	−	*De novo*	0
Fam16-1	74136215	c.412T>A	p.Y134N	−	*De novo*	0
Fam17-1	74129547	c.187C>G	p.R63G	+	Unknown	0
Fam19-4	74129825	c.330C>A^b^	p.F110L	−	Unknown	0
Fam25-1	74141962	c.769C>T	p.R257C	+	*De novo*	0
Fam26-1	74128449	c.118C>T	p.R40C	+	*De novo*	0
Fam28-1	74140753	c.593G>A	p.G198D	−	*De novo*	0
Fam29-1	74140692	c.532C>T	p.R178C	+	*De novo*	0
Fam30-1	74141962	c.769C>T	p.R257C	+	*De novo*	0
Fam34-1	74128450	c.119G>A	p.R40H	+	Paternal	0
Fam35-1	74140693	c.533G>A	p.R178H	+	*De novo*	0

a Deduced cDNA change in transcript NM_001615 unless otherwise indicated.

b Transcript Uc010fex.1.

c Deduced amino acid substitution.

d Presence of the observed mutation in other exomes from the Baylor Center for Mendelian Genomics cohort.

### Clinical Characteristics of Smooth Muscle Disease Due to *De Novo ACTG2* Mutations

The clinical characteristics of the patients with apparent *de novo* mutations are summarized in Table 3 and in Text S1. The age at the time of follow-up was from less than one year to 25 years. In the ten apparently *de novo* cases, seven patients were diagnosed with megacystis prenatally, and two of these underwent fetal surgery. The three individuals without megacystis were nonetheless dependent on catheterization of the bladder long-term. Prune-belly syndrome was observed in one of the cases (Fam16-1).

**Table 3:** Clinical characteristics of the patients with MMIHS due to *ACTG2 de novo* mutations.

Subject	Fam4-1	Fam12-1	Fam14-1	Fam16-1	Fam25-1	Fam26-1	Fam28-1	Fam29-1	Fam30-1	Fam35-1
*ACTG2* Mutation	p.R257C	p.R40H	p.M45T	p.Y133N	p.R257C	p.R40C	p.G198D	p.R178C	p.R257C	p.R178H
Gender	M	M	F	M	M	F	F	M	F	F
Age in years	11	16	18	25	13	16	3	3	1	6
Paternal age at birth	28	37	39	28	44	36	32	26	24	32
Maternal age at birth	31	33	35	26	36	33	31	32	26	28
Megacystis	−	+	−	+	+	+	−	+	+	+
Fetal bladder diversion	−	+	−	+	−	−	−	+	−	−
Neonatal bilious emesis	+	−	+	−	+	−	+	+	+	+
Abdominal surgery/malrotation	+	−	+	+	+	−	+	+	+	+
Microcolon	−	−	+	−	+	+	−	+	−	+
Lifetime TPN dependence	+	−	−	−	+	+	+	+	+	+
Lifetime bladder catheterization	+	+	−	+	−	+	+	+	+	+
Motility treatment*(response)	M (−) Cis(−)	Cis (+)	Cis (+)	Cis (+)		Cis (−)				M (−)E (−)
Other medical conditions	Non-febrile seizures age 3 y	ADHD since age 6 y		Asthma, pectus excavatum, prune belly. cardio-myopathy	ADHD			Undescended testicle		

+ Feature present, − Feature absent,

*M- Metaclopramide, Cis- Cisapride, E-Erythromycin, (+) responsive, (−) non-responsive.

The gastrointestinal manifestations were similarly severe. Of the ten apparent *de novo* cases, seven had bilious vomiting as a neonate, and eight were diagnosed with intestinal malrotation. All ten patients had multiple abdominal surgeries (Table 4). Long-term dependence on TPN was a consistent feature, but did not extend throughout life for all the patients. Two patients had very intermittent TPN requirements, usually during surgical recovery. Another patient (Fam26-1) had an interval of improvement at age four years followed by reinitiation of TPN at six years.

**Table 4:** Natural history of patients with MMIHS due to *ACTG2 de novo* mutations.

Subject	Fam4-1	Fam12-1	Fam14-1	Fam16-1	Fam25-1	Fam26-1	Fam28-1	Fam29-1	Fam30-1	Fam35-1
Mutation	p.R257C	p.R40H	p.M45T	p.Y133N	p.R257C	p.R40C	p.G198D	p.R178C	p.R257C	p.R178H
Surgeries/age	Vesicostomy/neonate	Hernia repair/neonate	Ladd surgery/neonate	Vesicostomy/neonate	Jejun-ostomy, ileostomy/1 y	Vesicostomy/neonate	Ladd surgery/neonate	Removal of bladder shunt/neonate	Small bowel ostomy/<1 y	Ladd surgery/neonate
	Malrotation/3 months	Ileostomy/1 y	Colon/ileum resection ileostomy/9 y	Ileostomy and colectomy/3 months	Ileostomy revision/1 y	Ileostomy/9 y	Rectal biopsy/neonate	Ileostomy/<1 y	Rectal biopsy/<1 y	
	Appendectomy/chole-cystectomy/3 months	Ileostomy revision/2 y	Hemi-gast-rectomy removal of pylorus/10 y	Pectus surgery, abdominal wall recon-struction/adolescent	Multi-visceral transplant/3 y	Colectomy/16 y	Lysis of adhesions/2 months			
Functional study	Manometry with absent peristalsis					Manometry with absent peristalsis	Manometry with absent peristalsis	Small bowel follow-through with delayed emptying		Manometry with absent peristalsis
Outcome	Death at 11 years from multiple bouts of pancreatitis	Ileostomy, catheter dependent cognitively normal	IV fluids and food, self-voiding, cognitively normal	Eating by mouth, urostomy, ileostomy, hypothyroid	Intestinal transplant, ileostomy, clinically well	Frequent infections listed for multi-visceral transplant	Partial parenteral nutrition, catheter dependent	TPN and catheter dependent, cognitively normal	TPN dependent, cognitively normal	TPN dependent, cognitively normal

Interestingly, of the ten apparent *de novo* patients, three reported partial but significant clinical improvement on cisapride, a serotonin 5-HT_4_ receptor agonist and gastroprokinetic agent (see Text S1). Recently this drug was removed from the market for cardiac side effects, but one patient continued on the drug as an FDA-approved case of compassionate use, and the two others indicated strong desires to remain on the drug despite the risks. Two other families found that the same drug did not have a significant effect.

Clinical outcomes in our cohort differ from the 19.7% survival rate reported in the literature [2]. Of the ten apparent *de novo* cases, nine were alive at the time of last follow-up; while one individual died at age 11 after multiple episodes of pancreatitis. One individual had undergone an intestinal transplant, and one individual was wait-listed for combined intestinal and liver transplant. Of the nine surviving individuals, eight had ileostomies. The oldest survivor amongst the apparent *de novo* cases was 25 years old, while the oldest previously reported case of MMIHS was 24 years [2]. While this suggests improved survival in our cohort, we observed frequent abdominal surgery, and dependence on TPN and chronic catheterization, suggesting that improvements in supportive care over time rather than a milder phenotype associated with *ACTG2* mutations are responsible for this improved survival.

### Familial Disease Due to *ACTG2* Mutations

In addition to these apparent *de novo* cases, five other probands were heterozygous for *ACTG2* variants; in three of these cases the mutation was inherited from one of the parents. In one case the inheritance remains unknown as parental samples are not available. In an additional family (Fam19), the proband and an affected sibling both carry a heterozygous mutation that affects an alternate exon 4 (c.330C>A; p.F110L) of a predicted *ACTG2* short isoform. The proband (Fam19-1) had multiple abdominal surgeries for obstruction and long-standing hypomotility. She has been on TPN intermittently since age 17 years, but has not required bladder catheterization. The sibling (Fam19-4) suffered years of intestinal symptoms and underwent an endoscopy suggesting gastroparesis. However she had not had any abdominal surgery. No parental clinical information was available. The mother does not carry this mutation, and a paternal sample is not available. The data from this family suggests but perhaps do not prove entirely that the alternative exon 4 which would result in a very short protein isoform is functionally important.

In all three inherited cases, we observed mutations identical to those identified in the apparent *de novo* cases (c.119G>A; p.R40H, c.533G>A; p.R178H and c.769C>T; p.R257C). The clinical findings in the familial cases and in the affected parents were notable for milder disease, more frequently classified as intestinal pseudo-obstruction. In one family (Fam34), the proband inherited the mutation (c.119G>A; p.R40H) from the father, who had no history of megacystis or neonatal abdominal surgery but had two abdominal surgeries in adulthood for episodes of gastrointestinal obstruction. Multiple paternal relatives over four generations carried the same mutation and were noted to have bowel and bladder dysfunction segregating as an autosomal dominant mutation (Figure 2).

In another family, the proband (Fam 13-1) inherited the mutation (c.769C>T; p.R257C) from the mother (Figure 2). The proband had prenatal megacystis but had a period of normal bowel and bladder function in the first years of life. She eventually experienced progressive pseudo-obstruction and ultimately died at age 13 years. Medical records were not available for her mother, but a history of gastrointestinal disease and a diagnosis of irritable bowel syndrome were reported.

### Predicted Functional Effect of the *ACTG2* Mutations

**Figure 3:**
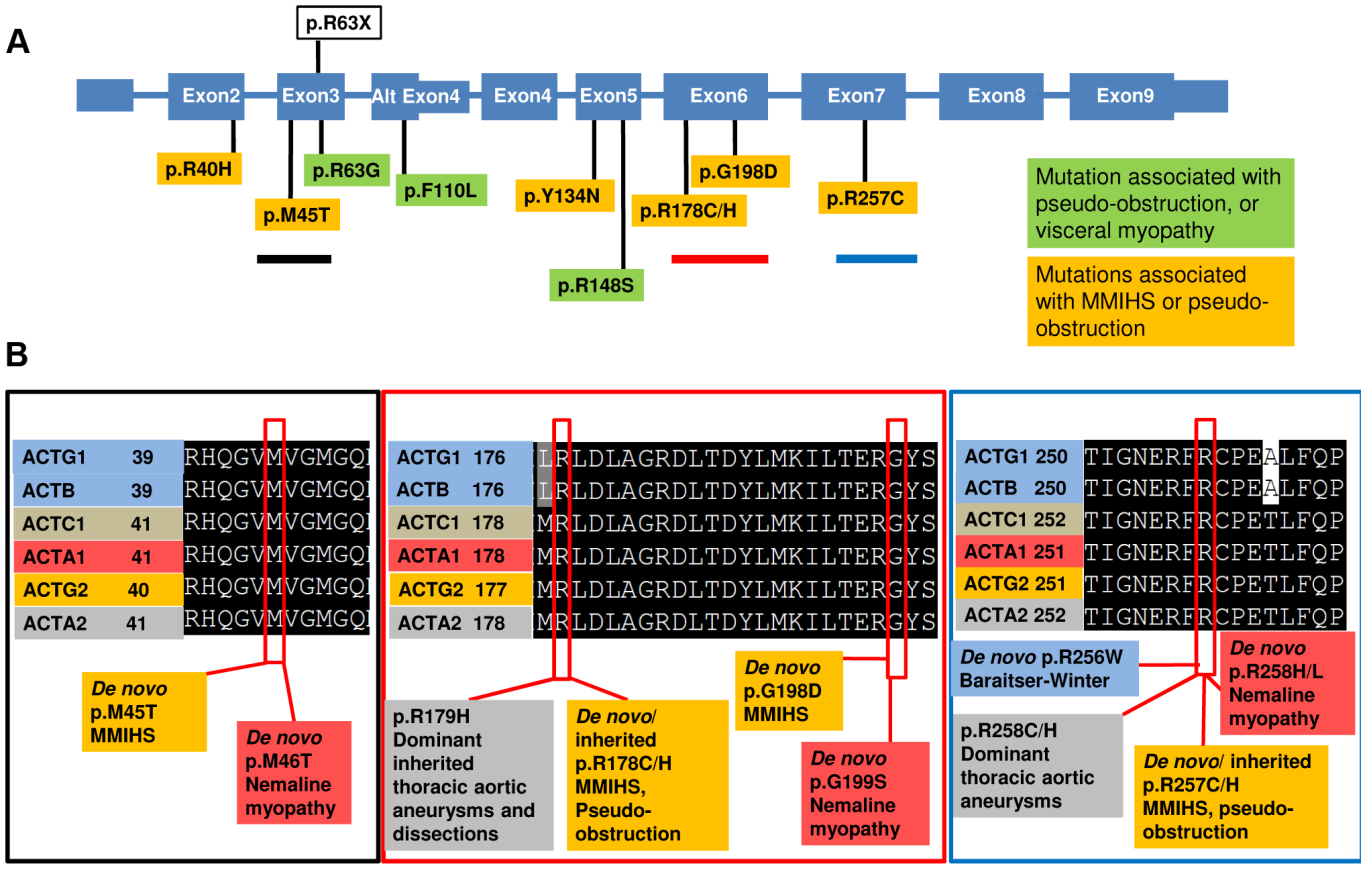
*ACTG2* mutations affect conserved residues that are also targets for Mendelian disease. A.) Depiction of the mutations on the exons of the gene. Introns are not shown to scale. The mutations associated with MMIHS and intestinal pseudo-obstruction (orange) and those associated with intestinal pseudo-obstruction (green), including the previously reported mutation in one Finnish family are shown. A nonsense allele at position R63 was identified in our exome database associated with no clinical phenotype. The black, red, and blue lines under specific mutations highlight areas of multi-sequence alignment in boxes of corresponding colors in B. B.) Comparison of the mutations in MMIHS/intestinal pseudo-obstruction with disease causing mutations in other actin genes.

The mutations we observed extended from exon 2 to exon 7 of the transcript (Figure 3A). Alignment of all six human actin proteins, which are highly conserved, revealed identity among all the amino acids in which we observed substitutions. All of these genes are implicated in human disease. *De novo* missense mutations in *ACTB* and *ACTG1* underlie the brain malformation syndrome Baraitser-Winter (OMIM 243310) [32], [33]. Mutations in *ACTC1* are implicated in a range of cardiac phenotypes including cardiomyopathy (OMIM 613424) [34], cases of nemaline myopathy (OMIM 161800) are due to mutations in *ACTA1* which can be dominantly or recessively inherited depending on the mutation [35], and *ACTA2* mutations are associated with incompletely penetrant dominantly inherited aneurysm and dissections (OMIM 611788). We compared the *ACTG2* mutations in our cohort to mutations from these disorders in the identical amino acid position along the actin filament (Figure 3B). There was clearly alignment between multiple mutations observed in our cohort and those implicated in Baraitser-Winter, nemaline myopathy, and thoracic aortic aneurysms and dissections.

We also explored the question of the phenotypic consequences of haploinsufficiency at *ACTG2*. We identified an incidental nonsense mutation (c.C187T:p.R63X) in *ACTG2* in our internal exome sequencing database from a group of approximately 1900 individuals in the Center for Mendelian Genomics at Baylor College of Medicine (Table S1). The individual was an unaffected parent from a study of an unrelated disorder. This individual reported mild intermittent constipation, not requiring medications, and no history of abdominal surgery, or bladder dysfunction. In aggregate, these data suggest that *ACTG2* haploinsufficiency is not clinically significant, although a mild phenotype with incomplete penetrance cannot be excluded. For all of the genotype-phenotype relationships shown in Figure 3B, virtually all of the reported mutations are missense with very few or no examples of frameshift or nonsense mutations. One reason for this bias might be that heterozygous loss-of-function mutations are benign or cause a different phenotype for these actin loci.

### CpG Dinucleotides in *ACTG2* and Paternal Age

**Figure 4:**
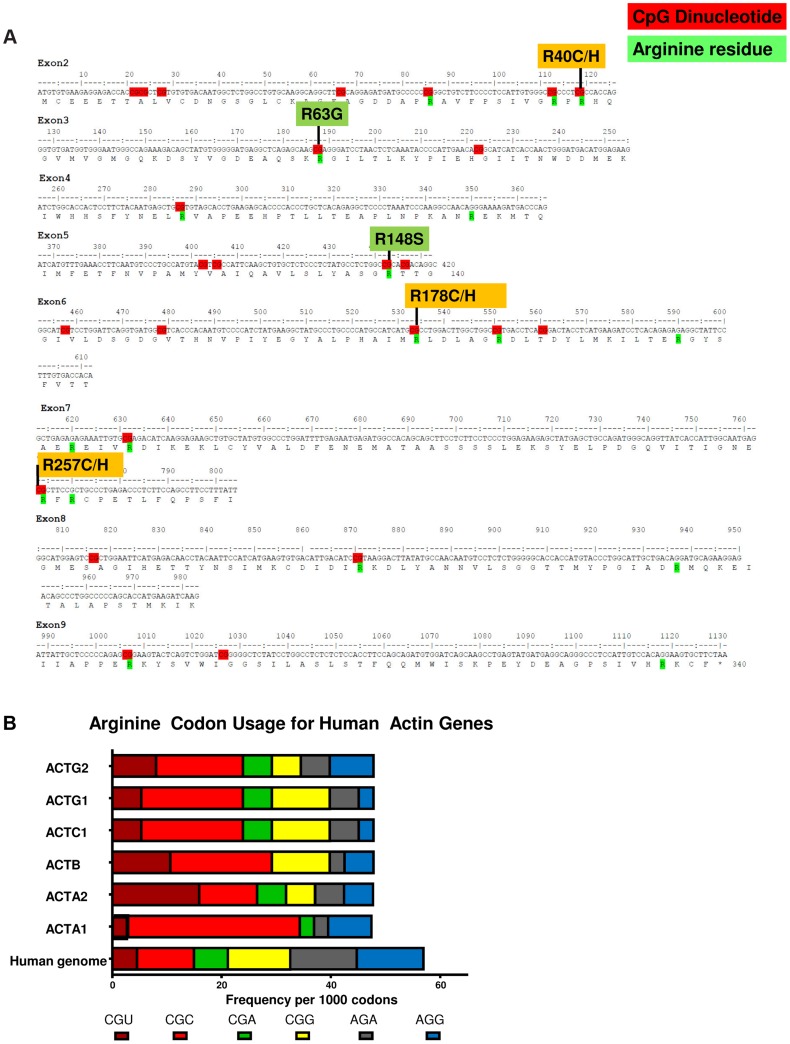
CpG dinucleotides within arginine codons are targets of *de novo* events in MMIHS. A.) The coding exons are shown with translation for the *ACTG2* gene. CpG dinucleotides are highlighted in red. Arginine residues in the protein are highlighted in green, and the mutations associated with *ACTG2* smooth muscle disease are aligned above the sequence. B.) The frequency of codon usage per 1000 codons for 6 arginine codons is shown. The human genome as a whole (bottom bar) is compared to all human actin genes including *ACTG2*.

Out of the 10 variants in *ACTG2* identified in 14 unrelated probands, six result from C>T transitions at CpG dinucleotides altering an arginine codon (Figure 4A). We observed that the CGC codon encodes 33% of the arginine residues in the γ-actin protein compared to 18% of arginine residues genome wide (Figure 4B) [36]. One explanation for the pattern of codon usage could relate to the expression of actin genes, as more highly expressed genes have been observed to have significantly skewed codon usage [37]. Given the presence of multiple CpG dinucleotides due to this pattern of codon usage, we surveyed paternal age in our *de novo* cases. We observed an average paternal age of 32.7 years amongst the families with *de novo* mutations with a standard deviation of 6.7 years which is not sufficient to conclude statistically whether these ten apparently *de novo* mutations may be associated with advanced paternal age.

## Discussion

Identification of *ACTG2* mutations underlying a significant proportion of MMIHS and intestinal pseudo-obstruction has significance for three major reasons. First, autosomal dominant rather than autosomal recessive mutations now are known to be present in the majority of families (15 of 26 probands reported in this study). Many cases in the literature as well as the Online Mendelian Inheritance in Man database suggest autosomal recessive inheritance. While other loci exhibiting recessive inheritance are possible, nearly half of our cases of MMIHS appear to follow a dominant or sporadic pattern of inheritance with heterozygous mutations segregating with the phenotype.

Second, the phenotypic spectrum for disease causing mutations in *ACTG2* can now be relatively well defined. All of the apparent *de novo* cases had clear indications of severe smooth muscle disease with prenatal or neonatal onset, urinary catheterization, and dependence on TPN. However, phenotypic variability and distinct complications existed such that prune belly syndrome, MMIHS, hollow visceral myopathy, and intestinal pseudo-obstruction were all diagnosed. Reorganizing these clinical entities into a spectrum of *ACTG2* related disorders will be of great benefit in understanding the natural course of these diseases.

Third, a better understanding of the pathophysiology could lead to treatment opportunities. It was notable that three individuals reported here with MMIHS due to apparent *de novo* mutations in *ACTG2* reported clinical improvement on cisapride, a serotonergic 5HT_4_ agonist. Serotonergic activity at the 5HT_4_ receptor has been noted to be a therapeutic target for constipation and irritable bowel syndrome [38]. Cisapride was proposed as a therapy for MMIHS in 1991 [14]. Subsequent case reports have noted failure of cisapride to produce clinical improvement in MMIHS [39], [40]. However in our cohort three patients out of five who had been prescribed cisapride reported improved motility by subjective clinical improvement. Two of the three patients were taken off this drug for cardiac side effects. Given that new 5HT_4_ agonist agents with safer cardiac profiles are under study [41], patients with *ACTG2* mutations may be candidates for this therapeutic strategy. Also, the mutational data suggest the feasibility of selective knock-down of the mutant transcript using antisense oligonucleotides, offering some therapeutic hope to affected individuals and families [42].

Finally our study effectively refocuses the study of MMIHS back to the contractile apparatus of the smooth muscle in an analogous way to how cardiomyopathy and myopathy tie to muscle contractile genes. Mendelian disorders of skeletal and cardiac muscle function have historically underscored the essential role of the sarcomere and its contractile apparatus in human health and disease [43], [44]. Since Huxley formulated the sliding filament model, all muscle contraction has been understood as a product of the interaction of two polymers, the thin filament actins and thick filament myosins [45]. Mendelian disorders largely conform to Huxley's fundamental insight as numerous disorders are now attributed to mutations in actins, myosins, and related proteins [46], [47]. Sarcomere proteins had not been previously explored in MMIHS perhaps because the role of sarcomeric proteins in smooth muscle disease is less clear than in skeletal and cardiac disorders, and smooth muscle lacks the rigid alignment of the sarcomeres seen in cardiac and skeletal muscle. However, vascular smooth muscle disease has also been attributed to mutations in actin and myosin genes with the discovery of mutations in *ACTA2* [48] and *MYH11* [49] in thoracic aortic aneurysms and dissections. There are also reports of a specific mutation in *ACTA2* associated with vascular aneurysms and hypomotility of the gastrointestinal tract (OMIM 613834) [50] and also with prune belly sequence [51]. Additionally, as mentioned above, adult onset visceral myopathy was recently associated with a dominant mutation in the *ACTG2* enteric actin gene in a Finnish family [20]. These findings provide the context for our data demonstrating a role for *ACTG2* in MMIHS. While the mouse model for *CHRNA3* generated promising insight into ganglion cell neurotransmission and smooth muscle function [27], the smooth muscle itself is clearly also involved in MMIHS. These results strongly suggest that there are other genes that are mutated in MMIHS, and candidate genes can be envisioned based on the combined data from mouse and human mutations. MMIHS can be considered the most severe Mendelian enteric smooth muscle myopathy.

After the submission of this manuscript, a report was published detailing the identification of *de novo ACTG2* mutations by exome sequencing in two children with MMIHS [52].

## Materials and Methods

### Study Subjects and Ethics Statement

Informed consent was obtained prior to participation from all subjects or parents of recruited subjects under one of two Institutional Review Board approved protocols at Baylor College of Medicine.

### Clinical Evaluation

Whenever possible, our clinicians assessed study subjects by direct history, physical examination, and family history analysis. In some cases, clinicians referred subjects from centers around the world, and in those cases clinical information in the form of chart records and notes from the referring physicians were reviewed. Interviews with these subjects were also conducted by telephone. Families were asked prenatal history, and dates and nature of abdominal surgeries. Whenever available, reports from prenatal ultrasound, operative reports, manometry, or radiologic studies were reviewed.

### Whole-Exome Capture and Sequencing

Methods utilized for whole-exome sequencing have been previously described in detail [53]. In summary, 1 µg of genomic DNA was fragmented by sonication in a Covaris plate (Covaris, Inc. Woburn, MA). Genomic DNA samples were constructed into Illumina paired-end libraries as described [53]. Pre-capture libraries were pooled together and hybridized in solution to the BCM-HGSC CORE exome capture design [54] (52 Mb, NimbleGen). Captured DNA fragments were sequenced on an Illumina HiSeq 2000 platform producing 9–10 Gb per sample and achieving an average of 90% of the targeted exome bases covered to an minimal depth of 20× or greater.

### Data Analysis

Produced sequence reads were mapped and aligned to the GRCh37 (hg19) human genome reference assembly using the HGSC Mercury analysis pipeline (http://www.tinyurl.com/HGSC-Mercury/). Variants were determined and called using the Atlas2 [55] suite to produce a variant call file (VCF) [56]. High-quality variants were annotated using an in-house developed suite of annotation tools [57].

### Sanger Sequencing

Primers were designed to encompass all the exons and intron-exon boundaries of the *ACTG2* gene using ExonPrimer (Tim Strom, http://ihg.gsf.de/ihg/ExonPrimer.html) and Primer3 [58]. Sanger reads were analyzed using LASERGENE Seqman software [59].

### Alignments and Analysis

Multiple sequence alignments were performed using Clustal Omega [60] and depicted using Boxshade. Arginine codon usage was determined using the Codon Usage Database and the countcodon program of Yazukazu Nakamura.

## Supporting Information

Table S1 Novel variants in the *ACTG2* gene within the Center for Mendelian Genomics data excluding the MMIHS cohort.
(DOCX)

Text S1 Clinical Case histories for cases of MMIHS due to ACTG2 mutations. A clinical narrative from birth to present is provided for each of the patients with the nature of the *ACTG2* mutation.
(DOCX)
