# Supplementary material for: Heterozygous De Novo and Inherited Mutations in the Smooth Muscle Actin (ACTG2) Gene Underlie Megacystis-Microcolon-Intestinal Hypoperistalsis Syndrome
Source: PLoS Genet. 2014 Mar 27;10(3):e1004258. doi: 10.1371/journal.pgen.1004258 (PMC3967950; doi:10.1371/journal.pgen.1004258)
Supplement: Table S1 — Novel variants in the ACTG2 gene within the Center for Mendelian Genomics data excluding the MMIHS cohort. (DOCX) [file pgen.1004258.s001.docx]

**Table S1 Novel variants in the *ACTG2* gene within the Center for Mendelian Genomics data excluding the MMIHS cohort**

| **Variant Position (hg19)**  **Chr2** | **cDNA change^a^** | **Amino-acid change ^c^** | **CpG** | **Individuals with variant out of 1900^d^** |
| --- | --- | --- | --- | --- |
| 74141982 | c.C789G | p.F263L | - | 2 (inherited variant) |
| 74128560 | c.122_123insC | p.H41fs | - | 1 |
| 74129805 | c.A310G **^b^** | p.N104D | - | 2 (inherited variant) |
| 74146680 | c.T1109C | p.I370T | - | 2 (inherited variant) |
| 74146568 | c.C997A | p.P333T | - | 1 |
| 74129547 | c.C187T | p.R63X | + | 1 |
| 74136213 | c.T398C | p.M133T | - | 1 |

**^a^** deduced cDNA change in transcript NM_001615 unless otherwise indicated **^b^** transcript Uc010fex.1

**^c^** deduced amino acid substitution **^d^**presence of the observed mutation in any of the exomes from the Baylor Center for Mendelian Genomics cohort
