# Supplementary material for: Heterozygous De Novo and Inherited Mutations in the Smooth Muscle Actin (ACTG2) Gene Underlie Megacystis-Microcolon-Intestinal Hypoperistalsis Syndrome
Source: PLoS Genet. 2014 Mar 27;10(3):e1004258. doi: 10.1371/journal.pgen.1004258 (PMC3967950; doi:10.1371/journal.pgen.1004258)
Supplement: Text S1 — Clinical Case histories for cases of MMIHS due to ACTG2 mutations. A clinical narrative from birth to present is provided for each of the patients with the nature of the ACTG2 mutation. (DOCX) [file pgen.1004258.s002.docx]

**Clinical Case Histories**

The clinical information was gathered from clinical evaluations, interviews with the parents, and review of medical records when available.

**Fam4-1- p.R257C *de novo* *ACTG2* mutation:**

The patient was the product of an uncomplicated pregnancy with a normal ultrasound at 20 weeks gestation. After birth, there was a 2 week hospital stay in the Neonatal Intensive Care Unit (NICU) for respiratory distress; an enlarged bladder was noted. At 3 months of age, the patient developed bilious vomiting and was readmitted for abdominal surgery related to intestinal malrotation. During that admission, a vesicostomy was placed, and appendectomy and cholecystectomy were also performed. Microcolon was not noted. Total parenteral nutrition (TPN) was required since 3 months of age when a diagnosis of megacystis-microcolon-intestinal hypoperistalsis syndrome (MMIHS) was made.

Throughout life the patient required TPN and bladder catheterization. The patient was prescribed metaclopramide and cisapride with no response to either medication. At age 3 years the patient had seizure activity and was prescribed anti-epileptics for several months, but this was discontinued and no seizures occurred after age 4 years. At age 10 years the patient developed chronic pancreatitis and was admitted to the hospital for pancreatitis approximately once a month thereafter. He was subsequently listed for a liver and small bowel transplant. He died related to infectious complications of a bout of pancreatitis at age 11 years prior to transplant.

**Fam12-1- p.R40H *de novo* *ACTG2* mutation:**

The patient is the product of a pregnancy complicated by fetal megacystis treated with prenatal bladder diversion at 23 weeks. The birth was at 31 weeks gestation. The infant was fed by mouth but had frequent constipation. At age 2 years, a colostomy was placed but the infant continued feeding by mouth; a diagnosis of hollow visceral myopathy was made.

The patient, now 16 years of age, has a history of attention deficit hyperactivity disorder (ADHD) since age 6 years and has had lifetime dependence on bladder catheterization. Manometry studies were performed at 2 and 4 years of age both times showing no peristaltic activity. Despite this, the patient has not been TPN dependent. The patient was on cisapride and noted a good response in his ability to tolerate meals. He was taken off the medication for concern about the prolongation of the QT interval.

**Fam14-1- p.M45T *de novo* *ACTG2* mutation:**

The patient is the product of an uncomplicated pregnancy. She developed bilious vomiting shortly after birth and was taken to the operating room for a Ladd procedure to correct malrotation. Microcolon and dilated portions of intestine were noted. Urinary catheterization was required. At that time a diagnosis of MMIHS was made. From birth until 19 months the patient required parenteral nutrition. At 19 months, she was able to tolerate sufficient enteral nutrition that IV nutrition could be stopped. At one month of life, the patient was started on cisapride. The patient has been on cisapride since that time with only brief periods of being taken off around the time of a number of abdominal surgeries. At age 9, the patient required a resection of colon and ileum and ileostomy placement, and at age 15 the patient was placed back on parenteral nutrition and had an open cholecystectomy, revision of her gastrostomy, hemigastrectomy, intestinal stricturoplasy, and ileostomy revision.

The patient is currently 18 years of age, clincally well, and continues on cisapride under a compassionate use indication.

**Fam16-1- p.Y133N *de novo* *ACTG2* mutation:**

The patient is the product of a pregnancy complicated by fetal abdominal mass later determined to be megacystis. Delivery was via Cesarean section and a bladder diversion was conducted prior to delivery. At birth, absent abdominal wall musculature was noted, and the patient had a vesicostomy and abdominal wall reconstruction at 3 months of life when a diagnosis of prune belly syndrome and MMIHS was made. There was no bilious vomiting at birth but the patient was placed on TPN for feeding intolerance. The patient had abdominal surgery for intestinal obstruction at age 6 months and again at 4 years at which time an ileostomy was placed. The patient was put on cisapride and was noted to have moderate improvement in tolerance of feeding, but the medication was stopped due to prolonged QT interval on electrocardiogram.

The patient is currently 25 years of age and currently dealing with a number of health issues, in addition to the MMIHS and prune belly syndrome. These include ventricular dysfunction, asthma, pectus excavatum requiring surgery, osteoporosis, and endocrine abnormalities.

**Fam25-1 p.R257C *de novo* *ACTG2* mutation:**

The patient is the product of a term gestation. The pregnancy was complicated by fetal megacystis and polyhydramnios with premature membrane rupture. The patient was placed on TPN from birth and underwent jejunostomy after being noted to have a microcolon. At that time a diagnosis of MMIHS was made. A month later, an episode of abdominal obstruction led to another abdominal surgery. The patient remained on TPN until age 7 years at which time he underwent a transplant of small bowel, stomach, pancreas and colon. The patient also has a history of ADHD and one episode of seizures.

The patient is currently clinically well at age 13 years with ileostomy and on a regimen of double voiding twice a day.

**Fam26-1 p.R40C *de novo* *ACTG2* mutation:**

The patient is the product of a term gestation. The pregnancy was complicated by megacystis and polyhydramnios. The patient underwent vesicostomy at birth. She was noted to have a microcolon and required TPN. A diagnosis of MMIHS was made at birth. She did not have a response to cisapride or other promotility agents. She did have a period of relative improvement from age 4 years to 13 years, when she was off TPN for several years. She had more complications after age 13 years including a perforation of bowel and multiple infections.

She is currently 16 years old and awaiting small bowel transplantation. She is on TPN for 12 hours a day and taking table food and fluids through a gastrostomy. She also requires bladder catheterization.

**Fam28-1 p.G198D *de novo* *ACTG2* mutation:**

The patient is the product of an uncomplicated pregnancy. She was born at term and developed bilious vomiting after birth. She underwent abdominal surgery for malrotation including a Ladd procedure. She was not noted to have a microcolon but had some dilated loops of intestine. A diagnosis of MMIHS was made at birth. She was started on metaclopramide without any effect. At 40 days of life she was no longer voiding and was started on a catheterization regimen.

She is currently 3 years of age with normal developmental milestones, and she has required TPN and bladder catheterization throughout. However, her TPN has been reduced to 14 hours a day, and she has developed some spontaneous voiding.

**Fam29-1 p.R178C *de novo* *ACTG2* mutation:**

The patient is the product of a pregnancy complicated by fetal megacystis. An in- utero bladder diversion was undertaken. A diagnosis of MMIHS was made prenatally. After birth the patient developed bilious vomiting and underwent abdominal surgery for malrotation. The patient has continued to require TPN and bladder catheterization.

The patient is currently 3 years of age with normal developmental milestones. He requires TPN 18 hours per day and catheterization every 4-6 hours. The patient also has a history of an undescended testicle.

**Fam30-1 p.R257C *de novo* *ACTG2* mutation:**

The patient is the product of a pregnancy complicated by polyhdramnios. Fetal megacystis was noted at the 35^th^ week, at which time a Cesarean delivery was performed for fetal distress. Ultrasound did not reveal evidence of a microcolon. The child was discharged on oral feeding but returned at age 4 months with distended abdomen and dilated segments of intestine. Abdominal surgery to relieve the intestinal obstruction was undertaken and a diagnosis of MMIHS was made.

The patient is currently age 1 year with normal developmental milestones and dependent on TPN and catheterization. A trial of promotility agents is being considered.

**Fam34-1 p.R40H inherited ACTG2 mutation:**

The patient is the product of a pregnancy complicated by fetal megacystis noted at 20 weeks. At birth the patient developed feeding intolerance and had abdominal surgery for malrotation. At that time a diagnosis of MMIHS was made. The proband is currently 1 year of age with normal developmental milestones and on oral feeds but requiring catheterization.

A number of family members were also noted to have the R40H mutation including the father of the proband, the paternal grandmother, and the paternal great-grandmother, and a paternal first cousin. The father reported no diagnosis of MMIHS but has had symptoms of constipation and dysmotility since infancy. He has had abdominal surgery in adulthood including an episode of suspected superior mesenteric artery occlusion and removal of surgical adhesions. The paternal grandmother reports a lifetime history of constipation and abdominal surgery in adulthood to remove a megacolon. The paternal great-grandmother likewise reports lifetime history of constipation.

**Fam35-1 p.R178H *de novo ACTG2* mutation**

The patient is the product of a term gestation. The pregnancy was complicated by fetal megacystis, and a prenatal diagnosis of MMIHS was made. After birth, the patient developed bilious vomiting and underwent a Ladd procedure for malrotation and a microcolon was noted. The patient has had feeding intolerance and has required TPN and bladder catheterization throughout life. The patient had been on erythromycin and metaclopramide without response.

The patient is currently 6 years old with normal development in kindergarten classes. She is on TPN 13 hours a day and requiring bladder catheterization.
